# Comparison of pharmacokinetics and safety between CE-fosphenytoin sodium, fosphenytoin sodium, and phenytoin sodium after intravenous and intramuscular administration in healthy volunteers

**DOI:** 10.3389/fphar.2023.1204075

**Published:** 2023-11-17

**Authors:** Xiaojiao Li, Min Wu, Jixuan Sun, Weili Jin, Lei Han, Jia Xu, Jingrui Liu, Hong Zhang, Jing Wang, Daidi Wang, Hanyi Zhang, Qing Zhang, Nini Liu, Yanhua Ding

**Affiliations:** ^1^ Phase I Clinical Trial Center, The First Hospital of Jilin University, Changchun, China; ^2^ Xi’an Xintong Pharmaceutical Research Co., Ltd., Xi’an, China

**Keywords:** pharmacokinetics, safety, CE-fosphenytoin sodium, fosphenytoin sodium, healthy volunteers

## Abstract

**Background:** Captisol^®^-enabled-fosphenytoin sodium (CE-fosphenytoin sodium) injection is a modified formulation of fosphenytoin sodium.

**Objective:** We aim to compare the intravenous and intramuscular bioavailability and safety between CE-fosphenytoin sodium, fosphenytoin sodium (Cerebyx^®^), and phenytoin sodium (intravenous injection only).

**Methods:** In pivotal study 1, 54 subjects were divided into three sequence groups that receive intravenous injection of 250 mg of phenytoin sodium equivalent (PE), CE-fosphenytoin sodium (T), or fosphenytoin sodium (R1) and 250 mg of phenytoin sodium (R2) in period 1. After a 14-day washout period, 36 subjects were randomized to two treatment sequence groups (T-R1 or R1-T, *n* = 18 per group) in period 2, in which the subjects who received R2 in period 1 were removed, those who received T in period 1 used R1 (T-R1), while those who previously received R1 used T (R1-T). In pivotal study 2, a single intramuscular dose of T (400 mg PE) or R1 (400 mg PE) was administered according to the individual sequential treatment assignment in each period. There was a washout (14 days) period before receiving the next period study drug.

**Results:** T and R1 have similar pharmacokinetic characteristics regarding total and free phenytoin, showing bioequivalence of both drugs in the intravenous and intramuscular administration. The geometric mean ratio was close to 1 (0.98–1.06). The AUC of total and free phenytoin in subjects who intravenously received T and R1 was very similar to those who received R2, although their C_max_ was lower than that of the subjects who received R2. Overall, treatment with T and R1 was safe and well-tolerated, without serious adverse events (SAEs) or grade III adverse events (AEs). With intravenous (i.v.) or intramuscular (i.m.) treatment, the incidence of drug-related AEs using T was similar to that using R1. Treatment with T and R1 had clearly superior tolerability than that with R2.

**Conclusion:** CE-fosphenytoin sodium is a promising substitute for fosphenytoin sodium.

**Clinical Trial Registration:** http://www.chinadrugtrials.org.cn/, CTR20202154 (11 November 2020).

## Highlights

1. CE-fosphenytoin sodium injection is a modified formulation of fosphenytoin sodium. The new formulation improved the solubility, stability, and delivery of fosphenytoin sodium while maintaining its pharmacokinetic properties.
2. CE-fosphenytoin sodium and fosphenytoin sodium have similar pharmacokinetic characteristics regarding total and free phenytoin, showing the bioequivalence of both drugs in the intravenous and intramuscular administration.
3. Treatment with CE-fosphenytoin sodium and fosphenytoin sodium had clearly superior tolerability than phenytoin sodium.

## 1 Introduction

Because phenytoin sodium is insoluble and its pH is high (pH 12), administration of this medication could cause pain, swelling, tissue necrosis, and other side effects at the injection site. Fosphenytoin sodium is a phosphate prodrug of phenytoin sodium. Unlike phenytoin, fosphenytoin sodium is water-soluble, and the pH of its solution is about 8.6–9.0. Fosphenytoin has been used as an emergency drug to treat convulsions, without the disadvantages of phenytoin (Aweeka et al., 1999; Ogutu et al., 2003; Inoue et al., 2013; Kim et al., 2015). However, in the treatment with fosphenytoin sodium, especially by intravenous injection, paresthesia and pruritus often occur (FDA, 2021a).

Clinical application of fosphenytoin sodium has been approved in several countries, including the US and EU (1990s), and later in Japan (2011) (Tanaka et al., 2013). Intravenous and intramuscular administration of fosphenytoin sodium causes rapid and complete conversion of fosphenytoin to phenytoin, with a half-life of about 15 min. Maximum plasma concentration of fosphenytoin could be achieved by intravenous infusion (at the end of infusion) and intramuscular injection (approximately 30 min after injection). Fosphenytoin is predominantly distributed in the human plasma because it is extensively bound (95%–99%) to plasma proteins, such as albumin. Both acid and alkaline phosphatases are involved in the conversion of fosphenytoin, but its conversion rate is independent of patients’ age, race, and gender (Boucher et al., 1989; Jamerson et al., 1990; Browne et al., 1996; Knapp and Kugler, 1998).

CE-fosphenytoin sodium injection is a modified formulation of fosphenytoin sodium. Captisol^®^ was developed as an investigational formulation of fosphenytoin sodium through a patented drug formulation technology to generate a novel anionically charged sulfobutyl ether β-cyclodextrin molecule which can improve the solubility, stability, and delivery of fosphenytoin sodium (Ueda et al., 1998; Ren et al., 2014; Ren et al., 2020). CE-fosphenytoin sodium was approved by the FDA in November 2020. CE-fosphenytoin sodium injection has several advantages as follows: providing solubilization of any small amounts of phenytoin that may be generated in the formulation during storage; reducing injection risk; improving the stability of fosphenytoin sodium injection, which allows storing of this medication at room temperature, rather than at 2°C–8°C; and keeping the pH value (7.8–8.2) of this medication closer to the physiological pH value, unlike the pH value (8.6–9.0) of the marketed fosphenytoin sodium injection.

The pharmacokinetics of fosphenytoin sodium injection could be affected by many factors, such as the narrow therapeutic index and large inter-patient pharmacokinetic variability of phenytoin; the variation of CYP2C9, which is one of the main enzymes involved in the metabolism of phenytoin; and the variation of HLA-B*1502, which is known to be related to an increased risk of life-threatening Stevens–Johnson syndrome (SJS) and toxic epidermal necrolysis (TEN) in response to phenytoin treatment. Therefore, carbamazepine, phenytoin, or its prodrug fosphenytoin cannot be used in HLA-B*15:02 positive patients, according to the recommendation of the Clinical Pharmacogenetics Implementation Consortium (CPIC). However, in the HLA-B*15:02 negative and CYP2C9 intermediate metabolizers, there is at least a 25% reduction at the starting maintenance dose and at least a 50% reduction for HLA-B*15:02 negative and CYP2C9 poor metabolizers, with subsequent maintenance doses adjusted based on therapeutic drug monitoring and response (Dean et al., 2012; Caudle et al., 2014; Karnes et al., 2021).

To better understand the pharmacokinetic characteristics of CE-fosphenytoin sodium, we performed a comparison of the intravenous and intramuscular bioavailability and safety between CE-fosphenytoin sodium and its reference products fosphenytoin sodium (Cerebyx^®^) and phenytoin sodium (only for intravenous study) at an equivalent dose. The HLA-B*15:02 negative subjects and those with CYP2C9 normal metabolizers (CYP2C9*1) were enrolled in this study to observe the reduction in occurrence of side effects.

## 2 Materials and methods

### 2.1 Study drugs

The test product in this study was CE-fosphenytoin sodium injection (100 mg PE/2 mL), manufactured by Chengdu Tongde Pharmaceutical Co., Ltd. (Sichuan, China). The first reference product was fosphenytoin sodium injection (Cerebyx^®^) (100 mg PE/2 mL), manufactured by Pharmacia and Upjohn Company LLC (New York, United States). The second reference product was phenytoin sodium injection (100 mg/2 mL), manufactured by West-Ward, a Hikma Company (Eatontown, United States). Both the test and reference products were provided by Xi’an Xintong Pharmaceutical Research Co., Ltd. (Xi’an, China).

### 2.2 Study subjects

The recruited healthy volunteers were from Jilin province, China. The main inclusion criteria for recruiting study subjects were as follows: healthy men and non-pregnant women aged 18–50 years; having a body mass index of 18–28 kg/m^2^ (body weight was ≥50 kg for men and 45 kg for women); having no clinically significant abnormal findings on a laboratory test, physical examination, and medical history during the period of screening; and having a genotype of CYP2C9*1 plus HLA-B*15:02 negative. The main exclusion criteria of the study subjects included the following: having a history of porphyria, pseudolymphoma, sino-atrial block, second- and third-degree A-V block, long QT syndrome, Adams–Stokes syndrome, lymphoma and Hodgkin’s disease, and epilepsy or sensory disorders; the subject’s estimated creatinine clearance less than or equal to 80 mL/min, which was calculated using the Cockcroft–Gault equation; having allergic response to fosphenytoin sodium, phenytoin sodium, other hydantoins, or excipients in the formulations; having a history of long-time smoking or alcohol and/or drug abuse; having received any medication within 28 days prior to the initial dose of the study drug or during the study; or having participated in a clinical trial of an investigational drug in the previous 3 months.

### 2.3 Study design

**FIGURE 1:**
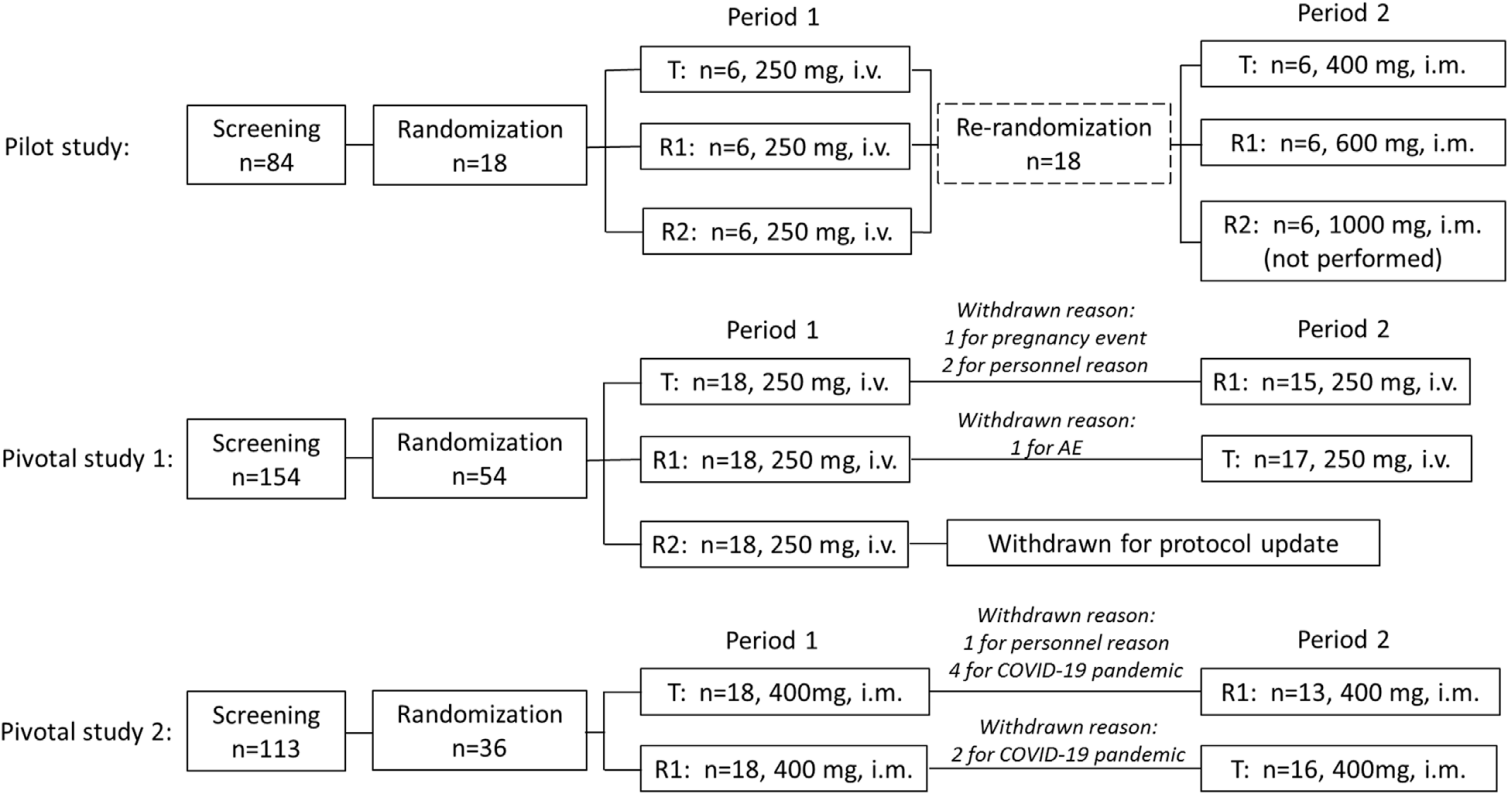
Study design and flow chart.

The flow chart of this clinical trial is shown in Figure 1, which includes the pilot study and two pivotal studies.

*Pilot study:* A total of 18 recruited subjects were randomly divided into three sequence groups (sequence 1, 2, and 3, *n* = 6 per group) in period 1. In each sequence, three experimental drugs were administered, respectively, including CE-fosphenytoin sodium (T, 250 mg PE, i.v.), fosphenytoin sodium (R1, 250 mg PE, i.v.), and phenytoin sodium (R2, 250 mg, i.v.). The infusion rate of those drugs was 40 mg PE/min for T and R1 and 40 mg/min for R2. After a 14-day washout period, in period 2, the aforementioned 18 subjects were re-divided into three sequence groups (*n* = 6, each). In each sequence, the subjects received a single dose of CE-fosphenytoin sodium (400 mg PE, 600 mg PE, and 1000 mg PE, successively) by intramuscular injection. After injection of 400 mg and 600 mg, an evaluation of the drug tolerance was performed, indicating that the incidence of 600 mg AEs was significantly higher than that of 400 mg PE. For safety considerations, the 1,000 mg PE dose group was not included.

*Pivotal study 1:* This was a single-dose, 3-treatment, 3-period, randomized, crossover study. A total of 54 recruited subjects were randomized to six treatment sequence groups (T-R1-R2, R1-R2-T, R2-T-R1, T-R2-R1, R2-R1-T, and R1-T-R2, *n* = 9 each). After the breakfast, subjects were administered (i.v.) with a single dose of T (250 mg PE), R1 (250 mg PE), or R2 (250 mg) according to their sequential treatment assignment in each period. There was a 14-day washout period before receiving the next period of study drugs. The infusion rates of the experimental drugs were 40 mg PE/min for T and R1 and 40 mg/min for R2. In addition, subsequent R2 administration was canceled for safety reasons due to the occurrence of intolerable adverse reactions in the R2 group in period 1. Therefore, modification of dosing design was needed. Accordingly, the study of three periods was changed to that of two periods, and the subjects who received R2 in period 1 were removed, the subjects who received T in period 1 were assigned to receive R1 in period 2 (T-R1), and the subjects who received R1 in period 1 were assigned to receive T in period 2 (R1-T).

*Pivotal study 2:* This was a single-dose, 2-treatment, 2-period, randomized, crossover study. A total of 36 recruited subjects were randomized to two treatment sequence groups (T-R1 or R1-T, *n* = 18 each). After breakfast, the subjects were administered (i.m.) with a single dose of T (400 mg PE) or R1 (400 mg PE), according to their sequential treatment assignment in each period. There was a 14-day washout period before receiving the next period study drug.

The protocol of this study was approved by the Ethics Committee of the Jilin University First Affiliated Hospital-Clinical Research Institute. This was a single-center clinical trial (the Jilin University First Affiliated Hospital-Phase I Clinical Research Center, Changchun City, China), with the clinical trial registration number CTR20202154 (http://www.chinadrugtrials.org.cn/, registration date: 11/Nov/2020), and following the international principles for clinical trials, such as the World Medical Congress Declaration of Helsinki and Good Clinical Practice guidelines, the US FDA and National Medical Products Administration (NMPA) Guideline for Bioequivalence Studies with Pharmacokinetic Endpoints for Generic Chemical Drugs, and the US FDA Guideline on Bioanalytical Method Validation. All study subjects provided written informed consent.

### 2.4 Analysis of PK profiles

The procedures for sample collection are described as follows. For i.v. administration (period 1 in the pilot study and pivotal study 1), blood samples were collected (by an indwelling intravenous angiocatheter and moved to the K_2_EDTA-containing tubes) at different time points, including at 0 h (before administration); at the end of the infusion; and 10, 20, 30, 40, and 50 min and 1, 1.5, 2, 3, 4, 6, 8, 12, 24, 48, 72, and 96 h after the start of infusion. For i.m. administration (period 2 in the pilot study and pivotal study 2), blood samples were collected at 0 h (before administration); 30 min; and 1, 2, 2.5, 3, 3.5, 4, 6, 8, 12, 24, 48, 72, and 96 h after dosing using the same collection method as mentioned previously. The first 0.5–1 mL of blood was discarded.

Blood samples (7 ml) collected at each time point were divided into two parts: 3 mL was used for separating the normal plasma by centrifugation at 2,600 *g* for 10 min at 4°C and then stored at −80°C, and the remaining 4 mL was used for separating the ultrafiltration plasma by centrifugation at 2,600 *g* for 10 min at 4°C and then (plasma) moved to an Amicon^®^ Ultra 4 mL Centrifugal Filter Unit (Merck KGaA, Darmstadt, Germany), which was placed in a centrifuge set at 25°C for 35 min within 1 h; after that, the ultrafiltration plasma was centrifuged again at 2,000 *g* for 60 min at 25°C. The obtained ultrafiltration plasma was stored in polypropylene tubes in two equal aliquots at −80°C until analysis.

The concentrations of total phenytoin were determined using a validated liquid chromatography with tandem mass spectrometry (LC-MS/MS) method. Shimadzu LC-30AD (Shimadzu Corporation, Kyoto, Japan) equipped with an LCMS-8050 triple quadrupole MS detector (Shimadzu Corporation, Kyoto, Japan) was used for analysis. The concentrations of free phenytoin were also determined by the LC-MS/MS method, but using another instrument, Shimadzu LC-20ADXR (Shimadzu Corporation, Kyoto, Japan) equipped with an LCMS-8060 triple quadrupole MS detector (Shimadzu Corporation, Kyoto, Japan). For total phenytoin and free phenytoin, the calibration ranges of the assays were 40.0–24,000 ng/mL and 5.00–3,000 ng/mL, respectively. The lower limits of quantification of human plasma using the two aforementioned detection methods were 40.0 ng/mL and 5.00 ng/mL, respectively. The accuracies of the detection methods for total phenytoin and free phenytoin were −4.2–0.9% and −4.0–1.6%, respectively. The precisions of the two methods were within 5.1 percent coefficient of variation (%CV) and 3.4% CV, respectively.

### 2.5 Safety assay

The safety and tolerability of the test drug and its reference products were evaluated, according to the National Cancer Institute Common Terminology Criteria for the Classification of Adverse Events (NCI-CTCAE, 5.0), based on the observation of the following aspects: AEs, vital signs (body temperature, blood pressure, and heart rate), 12-lead electrocardiograms, physical examination, clinical laboratory tests (such as biochemistry, hematology, urinalysis, coagulation, parathyroid hormone, and thyroid function), and injection site assessment. The recorded data of the incidence and severity of AEs and the relationship of the AEs to the study drugs were important documentation for safety evaluation.

### 2.6 Statistical analysis

The software WinNonlin^®^, version 8.2 (Certara, Princeton, NJ, United States), equipped with noncompartmental analysis methods, was used for analysis of PK profiles. Bioequivalence was established for free phenytoin, with bioequivalence assessments of total phenytoin providing supporting data. The parameters of PK profiles, such as C_max_, AUC_0-4_, AUC_0-t_, and AUC_0-∞_, were log-transformed, all of which were then analyzed using SAS 9.4 software. A mixed-effects model was used for calculating the geometric least squares means ratios (test/reference) and the corresponding 90% confidence intervals (CIs). The conclusion of bioequivalence could be drawn in the case of the 90% CI of the geometric mean ratios of C_max_, AUC_0-4_, AUC_0-t_, and AUC_0-∞_ between 80% and 125%. The Wilcoxon matched-pair signed-rank test was used for comparing the difference in T_max_ between the test and reference products.

## 3 Results

### 3.1 Baseline characteristics

A total of 351 subjects were screened, among which 18 subjects were enrolled (female, *n* = 8; male, *n* = 10) in the pilot study, 54 subjects (female, *n* = 27; male, *n* = 27) were enrolled in pivotal study 1, and 36 subjects (female, *n* = 14; male, *n* = 22) were enrolled in pivotal study 2. The demographic characteristics of the enrolled subjects are summarized in Table 1. In pivotal study 1, four subjects dropped out after period 1 administration due to pregnancy, personal reasons, or AE, including three subjects who received CE-fosphenytoin sodium and one subject who received fosphenytoin sodium. In pivotal study 2, seven subjects dropped out after period 1 administration due to the COVID-19 pandemic or personal reasons, including five subjects who received CE-fosphenytoin sodium and two subjects who received fosphenytoin sodium.

**TABLE 1:** Baseline demographic characteristics of the subjects enrolled in this study.

	Pilot study	Pivotal study 1	Pivotal study 2
	(N = 18)	(N = 54)	(N = 36)
Gender, N (%)			
Male	10 (55.6)	27 (50.0)	22 (61.1)
Female	8 (44.4)	27 (50.0)	14 (38.9)
Age (year)	37.22 ± 6.02	36.78 ± 7.94	37.58 ± 8.82
Race, N (%)			
Han	16 (88.9)	53 (98.1)	34 (94.4)
Other	2 (11.1)	1 (1.9)	2 (5.6)
Height (cm)	165.39 ± 7.74	163.71 ± 7.15	163.92 ± 8.70
Weight (kg)	62.82 ± 6.64	64.56 ± 8.70	65.76 ± 7.32
BMI (kg/m^2^)	22.94 ± 2.24	24.00 ± 2.08	24.53 ± 1.93

BMI, body mass index.

### 3.2 Pharmacokinetics

**FIGURE 2:**
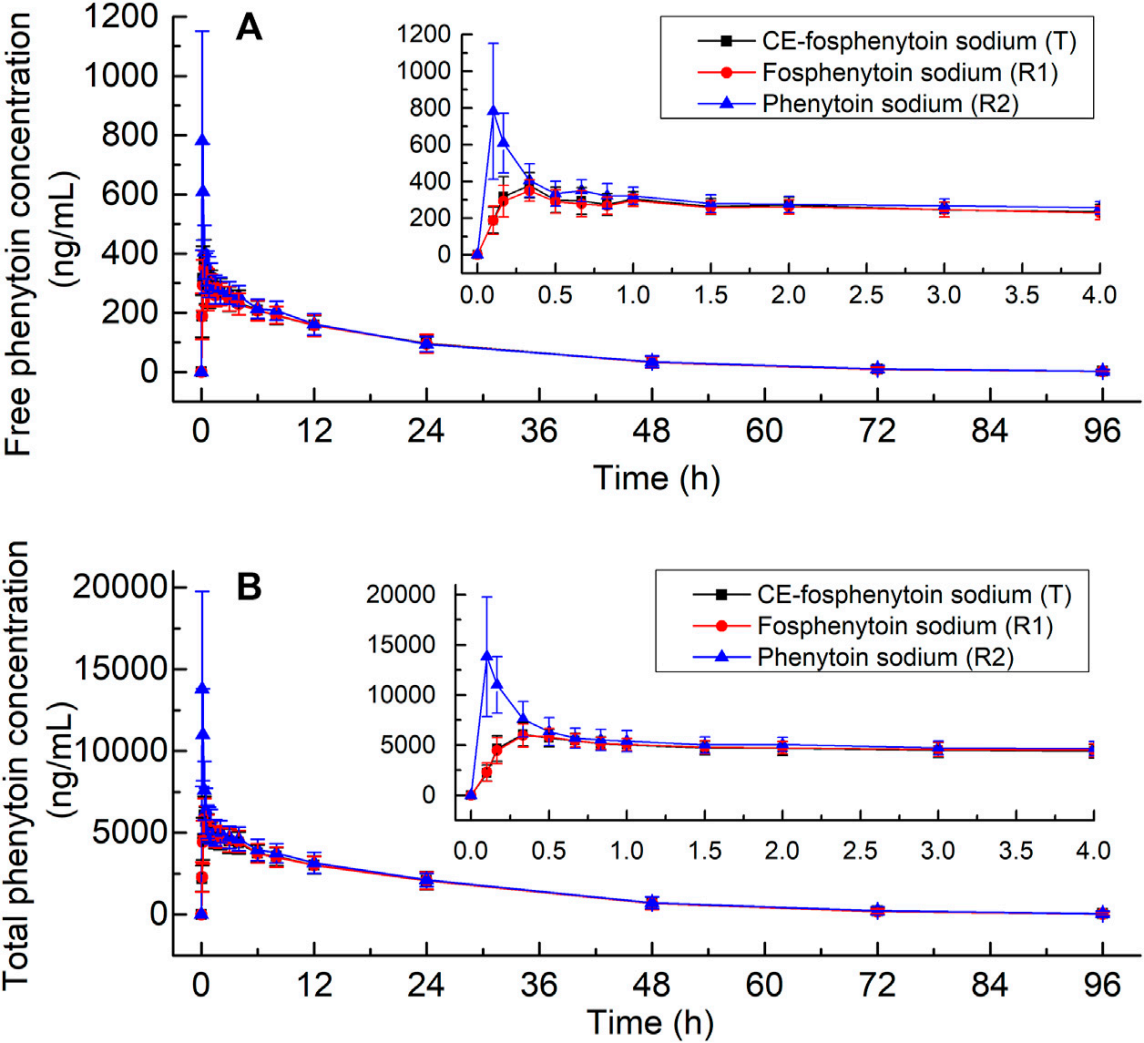
Mean values ± standard deviation of the plasma concentrations of free phenytoin **(A)** and total phenytoin **(B)** at different time-points after intravenous administration of CE-fosphenytoin sodium, fosphenytoin sodium, and phenytoin sodium in healthy subjects for pivotal study 1.

**FIGURE 3:**
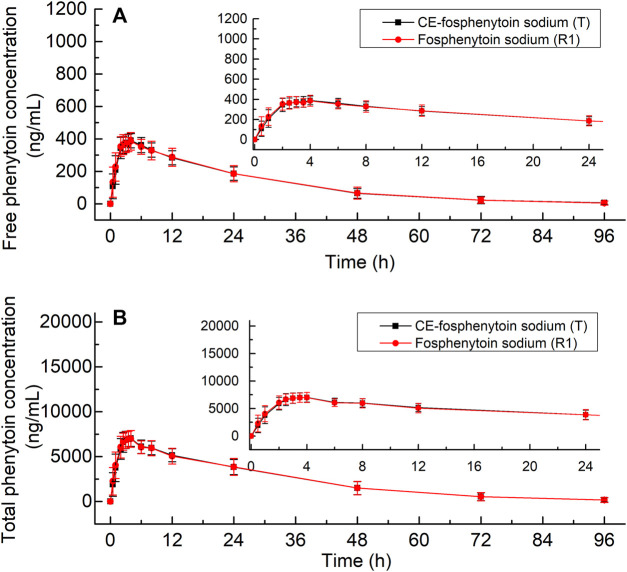
Mean values ± standard deviation of the plasma concentrations of free phenytoin **(A)** and total phenytoin **(B)** at different time-points after intramuscular administration of CE-fosphenytoin sodium and fosphenytoin sodium in healthy subjects for pivotal study 2.

The mean plasma concentrations of free and total phenytoin and their time profiles after the administration (i.v.) of 250 mg PE CE-fosphenytoin sodium, 250 mg PE fosphenytoin sodium, and 250 mg phenytoin sodium in pivotal study 1 are illustrated in Figure 2. The mean plasma concentrations of free and total phenytoin and their time profiles after administration (i.m.) of 400 mg PE CE-fosphenytoin sodium and fosphenytoin sodium in pivotal study 2 are illustrated in Figure 3. The mean plasma concentrations of free and total phenytoin were very similar after i.v. and i.m. administrations of CE-fosphenytoin sodium and fosphenytoin sodium.

The PK parameters of the administration (i.v.) of 250 mg PE CE-fosphenytoin sodium, 250 mg PE fosphenytoin sodium, and 250 mg phenytoin sodium in the pilot study (period 1) and pivotal study 1 are summarized in Table 2. The PK parameters of the drug administration in the pilot study were similar to those in the pivotal study. The values of C_max_ of CE-fosphenytoin sodium and fosphenytoin sodium were similar, which were lower than that of phenytoin sodium, but the values of the AUC of the three drugs as aforementioned were similar.

**TABLE 2:** Pharmacokinetic profiles of the intravenous administration study.

		T_max_* (h)	C_max_ (ng/mL)	AUC_0-t_ (ng∙h/mL)	AUC_0-4_ (ng∙h/mL)	AUC_0-∞_ (ng∙h/mL)	t_1/2_ (h)
**Free phenytoin**							
Pilot study (period 1)	T (N = 6)	0.42 (0.17,0.83)	388 (31.1)	5126.41 (11.36)	1002.08 (13.19)	5441.87 (11.75)	12.66 (9.79)
	R1 (N = 6)	0.33 (0.17,0.50)	377 (23.3)	5694.11 (25.98)	993.66 (16.57)	5985.40 (23.69)	13.49 (14.06)
	R2 (N = 6)	0.11 (0.10,0.12)	1090 (17.5)	6243.16 (12.22)	1200.66 (7.08)	6485.90 (11.71)	13.90 (15.29)
Pivotal study 1	T (N = 35)	0.33 (0.17,2.00)	391 (18.2)	6147.41 (27.03)	1059.42 (14.80)	6467.52 (27.60)	14.52 (28.50)
	R1 (N = 33)	0.33 (0.17,1.00)	368 (16.0)	6101.19 (29.41)	1032.77 (12.85)	6350.28 (29.11)	14.08 (18.68)
	R2 (N = 17)	0.11 (0.10,0.34)	818 (38.1)	6435.52 (26.88)	1213.69 (14.42)	6691.05 (28.29)	14.38 (19.12)
Simulated i.v., 30 min	R2	—	496	5848	—	—	—
**Total phenytoin**							
Pilot study (period 1)	T (N = 6)	0.42 (0.33,0.83)	6180 (23.5)	107852.94 (9.44)	18253.34 (12.86)	109256.61 (9.56)	11.59 (8.19)
	R1 (N = 6)	0.33 (0.33,0.50)	6030 (20.2)	121789.04 (26.75)	18419.53 (13.94)	123864.83 (26.71)	13.05 (16.20)
	R2 (N = 6)	0.11 (0.10,0.12)	18200 (9.66)	126641.65 (12.44)	21693.59 (5.11)	127866.72 (12.40)	12.64 (12.49)
Pivotal study 1	T (N = 35)	0.33 (0.17,0.67)	6240 (17.2)	124582.07 (24.71)	18664.50 (13.70)	129697.90 (28.08)	14.57 (34.44)
	R1 (N = 33)	0.33 (0.33,1.00)	6190 (15.7)	125121.85 (25.81)	18,862.58 (13.22)	127537.14 (26.61)	13.45 (19.82)
	R2 (N = 17)	0.10 (0.10,0.34)	14700 (33.0)	130453.20 (21.32)	21690.42 (14.53)	134622 (24.10)	14.01 (27.17)
Simulated i.v., 30 min	R2	—	9056	127,453	—	—	—

Data are presented as means (CV%) for all parameters, except for T_max_, which is median (range).

T_max_, time to maximum concentration; C_max_, maximum observed concentration; AUC_0-t_, area under the curve from 0 to last time of quantifiable concentration; AUC_0-4_, area under the curve from 0 to 4 h; AUC_0-∞_, area under the curve from the 0 to infinity time; and t_1/2_, terminal elimination half-life.

The PK parameters for i.m. administration of CE-fosphenytoin sodium for the pilot study (period 2) and pivotal study 2 are summarized in Table 3. An analysis of the relationship between exposure and drug dose showed that exposure increased proportionally between 400 mg and 600 mg. In the pivotal study, the Cmax and AUC of administrations (i.m.) of CE-fosphenytoin sodium and fosphenytoin sodium were similar. The values of Tmax and t1/2 were similar between free and total phenytoin, and the exposure was about 20 times that of free phenytoin.

**TABLE 3:** Pharmacokinetic profiles of the intramuscular administration study.

		T_max_* (h)	C_max_ (ng/mL)	AUC_0-t_ (ng·h/mL)	AUC_0-4_ (ng·h/mL)	AUC_0-∞_ (ng·h/mL)	t_1/2_ (h)
**Free phenytoin**							
Pilot study (period 2)	T 400 mg PE (N = 6)	3.00 (2.50,4.00)	352 (13.2)	9898.62 (18.82)	1057.97 (19.47)	10190.48 (18.84)	14.53 (12.37)
	T 600 mg PE (N = 6)	3.50 (2.50,6.00)	643 (13.5)	19200.47 (10.28)	1927.76 (25.95)	19505.24 (9.80)	13.51 (12.31)
Pivotal study 2	T (N = 34)	3.50 (2.00,6.00)	400 (11.6)	10907.02 (23.75)	1121.56 (19.19)	11232.19 (26.39)	14.72 (30.27)
	R1 (N = 31)	3.50 (2.00,6.00)	395 (13.1)	10909.46 (27.67)	1147.53 (18.93)	11320.43 (30.76)	14.92 (28.14)
**Total phenytoin**							
Pilot study (period 2)	T 400 mg PE (N = 6)	4.00 (2.50,4.00)	6670 (16.1)	199887.72 (17.81)	19403.82 (23.79)	201948.45 (18.07)	13.00 (6.95)
	T 600 mg PE (N = 6)	4.00 (2.00,8.00)	11500 (14.8)	381530.25 (10.88)	32565.40 (26.93)	387002.30 (11.34)	13.32 (19.50)
Pivotal study 2	T (N = 34)	3.50 (2.50,6.00)	7240 (12.4)	218007.51 (24.47)	20143.41 (19.15)	223454.61 (27.31)	14.61 (25.61)
	R1 (N = 31)	3.50 (2.00,4.01)	7200 (12.6)	217563.23 (25.83)	20687.85 (18.24)	224821.02 (30.58)	14.97 (34.16)

Data are presented as means (CV%) for all parameters, except for T_max_, which is median (range).

T_max_, time to maximum concentration; C_max_, maximum observed concentration; AUC_0-t_, area under the curve from 0 to last time of quantifiable concentration; AUC_0-4_, area under the curve from 0 to 4 h; AUC_0-∞_, area under the curve from the 0 to infinity time; and t_1/2_, terminal elimination half-life.

### 3.3 Bioequivalence

The bioequivalence of T and R1, which were administered (by i.v. and i.m.) in the pivotal study 1 and 2, was evaluated based on the crossover study, in which R1 was used as the reference drug. The results of the bioequivalence analysis (point estimates and corresponding 90% CIs) are summarized in Table 4. In regard to C_max_, AUC_0-t_, and AUC_0-∞_ of free and total phenytoin, the 90% CIs of the ratio of the T and R1 (administered by i.v. and i.m.) were found to meet the requirement of bioequivalence (within the 80%–125% range). In period 1 of pivotal study 1 (administered by i.v.), the bioequivalence of T and R2 and R1 and R2 was evaluated based on the results of a parallel study of the 18 subjects in each group, in which R2 was used as the reference drug. The results of the bioequivalence analysis (point estimates and corresponding 90% CIs) are summarized in Table 5. The 90% CIs of the ratio of the T and R2 for AUC_0-4_, AUC_0-t_, and AUC_0-∞_ of free and total phenytoin met the requirement of bioequivalence (within the 80%–125% range). Except for the 90% CIs of the ratio of R1 and R2 for free phenytoin AUC_0-t_ being 79.58%–105.89%, the other 90% CIs of the ratio of R1 and R2 for AUC_0-4_, AUC_0-t_, and AUC_0-∞_ of free and total phenytoin all met the requirement of bioequivalence (within the range from 80% to 125%). The C_max_ of T and R1 were lower than that of R2, which did not need transformation in the body.

**TABLE 4:** Point estimates and 90% CIs for the assessment of bioequivalence between T and R1 in the intravenous and intramuscular administration study (pivotal study 1 and 2).

Parameters	I.V.			I.M.		
	(T/R1)%	90% CI (%)	Intra-subject CV%	(T/R1)%	90% CI (%)	Intra-subject CV%
**Free phenytoin**						
C_max_ (ng/mL)	105.55	99.73–111.71	13.68	102.37	100.20–104.58	4.81
AUC_0-t_ (ng·h/mL)	100.00	96.67–103.44	7.87	101.61	99.09–104.19	5.60
AUC_0-4_ (ng·h/mL)	102.01	100.58–103.47	3.33	98.43	93.86–103.22	10.82
AUC_0-∞_(ng·h/mL)	100.00	96.99–103.09	7.09	101.31	98.68–104.00	5.86
**Total phenytoin**						
C_max_ (ng/mL)	99.67	94.98–104.58	11.53	99.94	97.53–102.40	5.47
AUC_0-t_ (ng·h/mL)	99.15	95.94–102.47	7.66	99.84	97.59–102.15	5.09
AUC_0-4_ (ng·h/mL)	98.65	96.96–100.36	4.07	96.47	92.48–100.64	9.55
AUC_0-∞_(ng·h/mL)	99.34	96.12–102.66	7.65	99.76	97.40–102.18	5.34

C_max_, maximum observed concentration; AUC_0-t_, area under the curve from 0 to last time of quantifiable concentration; AUC_0-4_, area under the curve from 0 to 4 h; and AUC_0-∞_, area under the curve from the 0 to infinity time.

**TABLE 5:** Point estimates and 90% CIs for assessment of bioequivalence between T and R2 or R1 and R2 in the intravenous administration study (pivotal study 1).

Parameters	T vs. R2		R1 vs. R2	
	(T/R2)%	90% CI (%)	(R1/R2)%	90% CI (%)
**Free phenytoin**				
C_max_ (ng/mL)	51.57	44.38–59.92	48.26	41.53–56.08
AUC_0-t_ (ng·h/mL)	94.08	81.39–108.74	91.79	79.58–105.89
AUC_0-4_ (ng·h/mL)	92.40	85.43–99.94	87.36	80.76–94.48
AUC_0-∞_(ng·h/mL)	94.30	81.59–108.99	92.65	80.33–106.86
**Total phenytoin**				
C_max_ (ng/mL)	46.51	40.28–53.70	44.17	38.25–51.00
AUC_0-t_ (ng·h/mL)	95.47	83.56–109.08	94.25	82.64–107.48
AUC_0-4_ (ng·h/mL)	88.68	82.18–95.70	86.49	80.15–93.33
AUC_0-∞_(ng·h/mL)	94.80	82.53–108.90	93.39	81.46–107.07

C_max_, maximum observed concentration; AUC_0-t_, area under the curve from 0 to last time of quantifiable concentration; AUC_0-4_, area under the curve from 0 to 4 h; and AUC_0-∞_, area under the curve from the 0 to infinity time.

### 3.4 Safety and tolerability

Safety data of all subjects during the period of this study, including all the subjects who received at least one dose of the study drug, were collected. Treatment-related adverse events that occurred in the intravenous administration group are summarized in Table 6, in which there were seven subjects (38.9%) with a total of 12 AEs in period 1 of the pilot study. Among those AEs, the incidence of drug-related AEs was 38.9% (7/18), in which two subjects in the treatment group of CE-fosphenytoin sodium had three drug-related AEs, with an incidence of 33.3% (2/6). No adverse events occurred in the fosphenytoin sodium-treatment group. A total of five subjects in the phenytoin sodium-treatment group had nine drug-related AEs (83.3% (5/6)). The incidence of drug-related AEs in the phenytoin sodium-treatment group was higher than that in the CE-fosphenytoin sodium- and fosphenytoin sodium-treatment groups. Treatment-related adverse events that occurred in the intramuscular administration group are summarized in Table 7 In period 2 of the pilot study, there were nine subjects (75.0%) with a total of 34 AEs, in which the incidence of drug-related AEs was 75% (9/12). Among those AEs, four subjects in the treatment group with 400 mg CE-fosphenytoin sodium had four drug-related AEs, with an incidence of 66.7% (4/6). A total of five subjects in the treatment group with 600 mg CE-fosphenytoin sodium had 29 drug-related AEs (83.3% (5/6)). The incidence of drug-related AEs in the treatment group administered with 600 mg CE-fosphenytoin sodium was higher than that in the group administered with 400 mg CE-fosphenytoin sodium. There were no SAE or grade III AEs in the pilot study.

**TABLE 6:** Treatment-related adverse events that occurred in the intravenous administration study.

	Pilot study			Pivotal study 1		
	T (N = 6)	R1 (N = 6)	R2 (N = 6)	T (N = 35)	R1 (N = 33)	R2 (N = 18)
All AEs	2 (33.3)	0 (0.0)	5 (83.3)	13 (37.1)	13 (39.4)	18 (100.0)
AE grade						
Grade I	2 (33.3)	0 (0.0)	3 (50.0)	12 (34.3)	12 (36.4)	14 (77.8)
Grade II	1 (16.7)	0 (0.0)	5 (83.3)	8 (22.9)	5 (15.2)	18 (100.0)
Grade III	0 (0.0)	0 (0.0)	0 (0.0)	0 (0.0)	0 (0.0)	1 (5.6)
Drug-related AEs	2 (33.3)	0 (0.0)	5 (83.3)	12 (34.3)	13 (39.4)	18 (100.0)
Injection site pain	0 (0.0)	0 (0.0)	5 (83.3)	2 (5.7)	1 (3.0)	18 (100.0)
Pruritus	1 (16.7)	0 (0.0)	1 (16.7)	6 (17.1)	5 (15.2)	1 (5.6)
Dizziness	0 (0.0)	0 (0.0)	1 (16.7)	3 (8.6)	3 (9.1)	3 (16.7)
Hypesthesia	0 (0.0)	0 (0.0)	0 (0.0)	0 (0.0)	0 (0.0)	8 (44.4)
Vertigo	0 (0.0)	0 (0.0)	0 (0.0)	3 (8.6)	1 (3.0)	4 (22.2)
Blurry vision	0 (0.0)	0 (0.0)	0 (0.0)	2 (5.7)	2 (6.1)	3 (16.7)
Headache	0 (0.0)	0 (0.0)	0 (0.0)	3 (8.6)	0 (0.0)	2 (11.1)
Urinary tract infection	0 (0.0)	0 (0.0)	0 (0.0)	1 (2.9)	3 (9.1)	1 (5.6)
Abdominal pain	0 (0.0)	0 (0.0)	0 (0.0)	1 (2.9)	1 (3.0)	2 (11.1)
Hidrosis	0 (0.0)	0 (0.0)	0 (0.0)	1 (2.9)	2 (6.1)	1 (5.6)
Nausea	0 (0.0)	0 (0.0)	0 (0.0)	3 (8.6)	0 (0.0)	0 (0.0)
Appetite decrease	0 (0.0)	0 (0.0)	0 (0.0)	2 (5.7)	0 (0.0)	1 (5.6)
Hypertriglyceridemia	0 (0.0)	0 (0.0)	0 (0.0)	0 (0.0)	1 (3.0)	1 (5.6)
Drowsiness	0 (0.0)	0 (0.0)	0 (0.0)	1 (2.9)	1 (3.0)	0 (0.0)
Joint pain	0 (0.0)	0 (0.0)	0 (0.0)	1 (2.9)	0 (0.0)	1 (5.6)
Lacking in strength	0 (0.0)	0 (0.0)	0 (0.0)	0 (0.0)	1 (3.0)	1 (5.6)
Diarrhea	0 (0.0)	0 (0.0)	0 (0.0)	1 (2.9)	0 (0.0)	1 (5.6)
Hypoglycemia	0 (0.0)	0 (0.0)	0 (0.0)	0 (0.0)	0 (0.0)	1 (5.6)
Asymptomatic bacteriuria	0 (0.0)	0 (0.0)	0 (0.0)	0 (0.0)	0 (0.0)	1 (5.6)
Elevated γ-GGT	0 (0.0)	0 (0.0)	0 (0.0)	1 (2.9)	0 (0.0)	0 (0.0)
Elevated ALT	0 (0.0)	0 (0.0)	0 (0.0)	1 (2.9)	0 (0.0)	0 (0.0)
Elevated AST	0 (0.0)	0 (0.0)	0 (0.0)	1 (2.9)	0 (0.0)	0 (0.0)
Parageusia	0 (0.0)	0 (0.0)	0 (0.0)	0 (0.0)	0 (0.0)	1 (5.6)
Dysgeusia	0 (0.0)	0 (0.0)	0 (0.0)	0 (0.0)	0 (0.0)	1 (5.6)
Allotriosmia	0 (0.0)	0 (0.0)	0 (0.0)	0 (0.0)	0 (0.0)	1 (5.6)
Tremors	0 (0.0)	0 (0.0)	0 (0.0)	1 (2.9)	0 (0.0)	0 (0.0)
Musculoskeletal discomfort	0 (0.0)	0 (0.0)	0 (0.0)	0 (0.0)	0 (0.0)	1 (5.6)
Limb pain	0 (0.0)	0 (0.0)	0 (0.0)	0 (0.0)	0 (0.0)	1 (5.6)
Penis pain	0 (0.0)	0 (0.0)	0 (0.0)	1 (2.9)	0 (0.0)	0 (0.0)
Non-infectious gingivitis	0 (0.0)	0 (0.0)	0 (0.0)	0 (0.0)	1 (3.0)	0 (0.0)
Dry mouth	0 (0.0)	0 (0.0)	0 (0.0)	0 (0.0)	1 (3.0)	0 (0.0)
Emesis	0 (0.0)	0 (0.0)	0 (0.0)	1 (2.9)	0 (0.0)	0 (0.0)
Palpitations	0 (0.0)	0 (0.0)	0 (0.0)	0 (0.0)	1 (3.0)	0 (0.0)
Dry eye	0 (0.0)	0 (0.0)	0 (0.0)	1 (2.9)	0 (0.0)	0 (0.0)
Ophthalmodynia	0 (0.0)	0 (0.0)	0 (0.0)	0 (0.0)	0 (0.0)	1 (5.6)
Purpura	0 (0.0)	0 (0.0)	1 (16.7)	0 (0.0)	0 (0.0)	0 (0.0)
Sinus bradycardia	1 (16.7)	0 (0.0)	1 (16.7)	0 (0.0)	0 (0.0)	0 (0.0)
Hypotension	1 (16.7)	0 (0.0)	0 (0.0)	0 (0.0)	0 (0.0)	0 (0.0)

AE, adverse event; data are presented as n (%): the number of subjects who developed any AEs (the incidence of the AEs).

**TABLE 7:** Treatment-related adverse events that occurred in the intramuscular administration study.

	Pilot study		Pivotal study 2	
	T (400 mg PE) (N = 6)	T (600 mg PE) (N = 6)	T (400 mg PE) (N = 34)	R1 (400 mg PE) (N = 31)
All AEs	4 (66.7)	5 (83.3)	12 (35.3)	14 (45.2)
AE grade				
Grade I	3 (50.0)	5 (83.3)	11 (32.4)	13 (41.9)
Grade II	1 (16.7)	3 (50.0)	0 (0.0)	1 (3.2)
Grade III	0 (0.0)	0 (0.0)	1 (2.9)	0 (0.0)
Drug-related AEs	4 (66.7)	5 (83.3)	11 (32.4)	14 (45.2)
Dizziness	2 (33.3)	5 (83.3)	6 (17.6)	5 (16.1)
Pruritus	0 (0.0)	4 (66.7)	3 (8.8)	3 (9.7)
Vertigo	0 (0.0)	0 (0.0)	1 (2.9)	2 (6.5)
Dizziness	0 (0.0)	0 (0.0)	1 (2.9)	2 (6.5)
Hypertriglyceridemia	0 (0.0)	0 (0.0)	1 (2.9)	1 (3.2)
Headache	0 (0.0)	3 (50.0)	1 (2.9)	1 (3.2)
Urinary tract infection	0 (0.0)	0 (0.0)	1 (2.9)	0 (0.0)
Increased white blood cell count	0 (0.0)	0 (0.0)	0 (0.0)	1 (3.2)
Elevated ALT	0 (0.0)	0 (0.0)	0 (0.0)	1 (3.2)
Elevated AST	0 (0.0)	0 (0.0)	0 (0.0)	1 (3.2)
Elevated serum creatinine	0 (0.0)	0 (0.0)	0 (0.0)	1 (3.2)
Increased neutrophil count	0 (0.0)	0 (0.0)	0 (0.0)	1 (3.2)
Hypesthesia	0 (0.0)	0 (0.0)	1 (2.9)	0 (0.0)
Ataxia	0 (0.0)	0 (0.0)	0 (0.0)	1 (3.2)
Hidrosis	0 (0.0)	3 (50.0)	0 (0.0)	1 (3.2)
Hematuria	0 (0.0)	0 (0.0)	0 (0.0)	1 (3.2)
Nausea	0 (0.0)	2 (33.3)	0 (0.0)	1 (3.2)
Abdominal discomfort	0 (0.0)	0 (0.0)	0 (0.0)	1 (3.2)
Abdominal pain	0 (0.0)	0 (0.0)	1 (2.9)	0 (0.0)
Sinus bradycardia	0 (0.0)	1 (16.7)	0 (0.0)	1 (3.2)
Dry eye	0 (0.0)	0 (0.0)	0 (0.0)	1 (3.2)
Oropharyngeal pain	0 (0.0)	1 (16.7)	0 (0.0)	0 (0.0)
Rash	0 (0.0)	1 (16.7)	0 (0.0)	0 (0.0)
Lacking in strength	0 (0.0)	3 (50.0)	0 (0.0)	0 (0.0)
Injection site pain	1 (16.7)	1 (16.7)	0 (0.0)	0 (0.0)
Diarrhea	1 (16.7)	0 (0.0)	0 (0.0)	0 (0.0)
Upper abdominal pain	0 (0.0)	1 (16.7)	0 (0.0)	0 (0.0)
Palpitations	0 (0.0)	1 (16.7)	0 (0.0)	0 (0.0)
Ophthalmodynia	0 (0.0)	4 (66.7)	0 (0.0)	0 (0.0)

AE, adverse event; data are presented as n (%): the number of subjects who developed any AEs (the incidence of the AEs).

In pivotal study 1, there were 37 subjects (68.5%) with a total of 119 AEs. Of these, the incidence of drug-related AEs was 66.7% (36/54). Among those AEs, 12 subjects in the CE-fosphenytoin sodium treatment group had 38 drug-related AEs, with an incidence of 34.3% (12/35). A total of 13 subjects in the fosphenytoin sodium group had 22 drug-related AEs (39.4% (13/33)). A total of 18 subjects in the phenytoin sodium-treatment group had 50 drug-related AEs (100% (18/18)). All of the 18 subjects in the phenytoin sodium-treatment group experienced infusion-site pain, most of which was accompanied by dizziness and numbness, and one subject had headache (grade III). However, the incidence rates for these AEs were much lower in CE-fosphenytoin sodium and fosphenytoin sodium-treatment groups. The incidence of drug-related AEs in the CE-fosphenytoin sodium group was a little lower than that in the fosphenytoin sodium-treatment group.

In pivotal study 2, there were 22 subjects (61.1% (22/36)) with a total of 43 AEs. Among those AEs, the incidence of drug-related AEs was 58.3% (21/36), in which 11 subjects in the CE-fosphenytoin sodium-treatment group had 15 drug-related AEs, with an incidence of 32.4% (11/34). A total of 14 subjects in the fosphenytoin sodium treatment group had 26 drug-related AEs (45.2% (14/31)). The incidence of drug-related AEs in the CE-fosphenytoin sodium group was similar to that in the fosphenytoin sodium-treatment group. There were no SAEs or grade III AEs in the administration (i.m.) groups.

**FIGURE 4:**
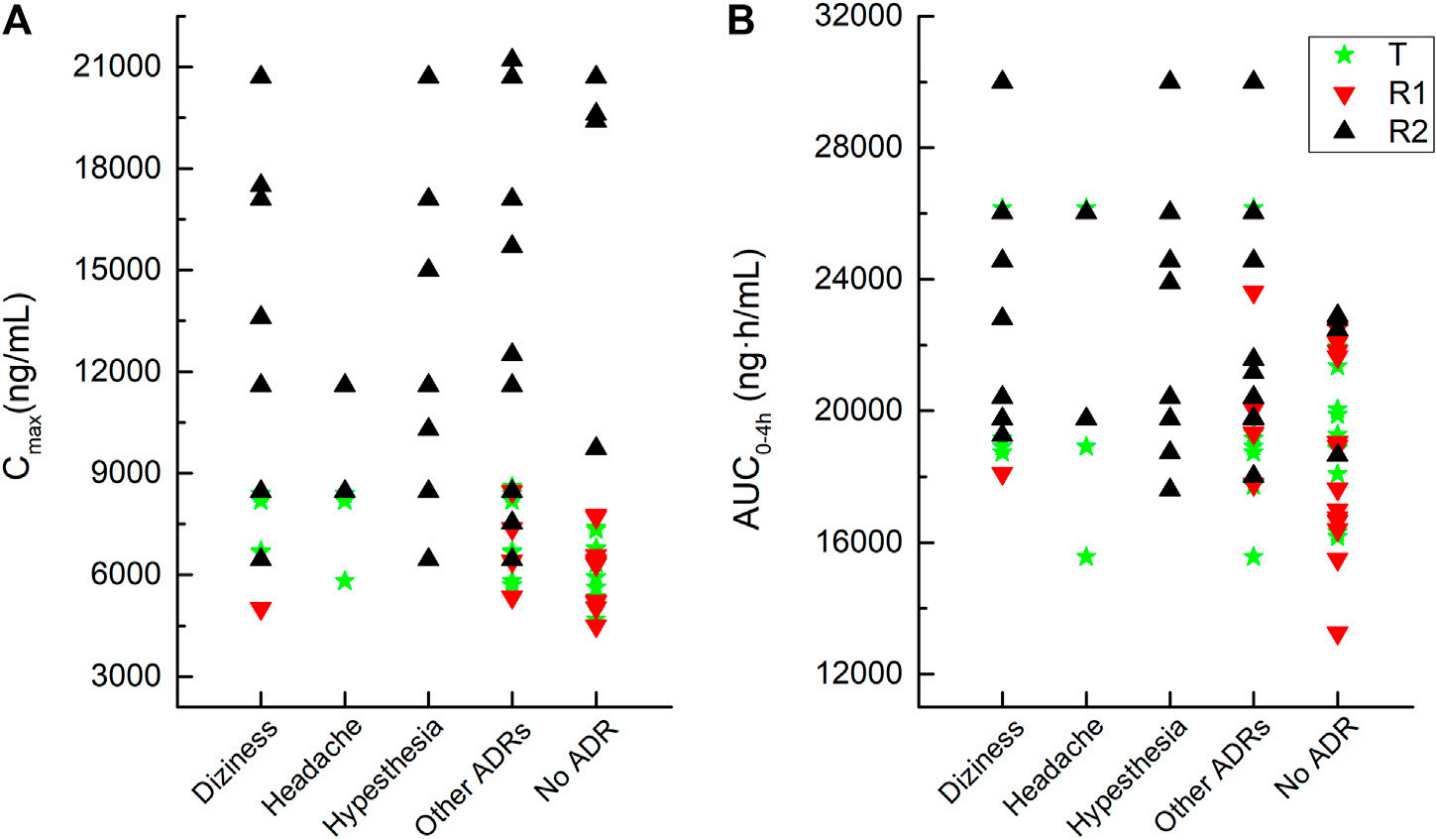
Correlation between adverse reactions and the values of the C_max_ and AUC_0–4h_ of plasma total phenytoin after intravenous administration of CE-fosphenytoin sodium, fosphenytoin sodium, and phenytoin sodium for pivotal study 1.

As shown in Figure 4, the C_max_ and AUC_0–4h_ of the plasma total phenytoin were visualized by the corresponding C_max_ and AUC_0–4h_ plot vs. the individual drug-related AEs that occurred in the pivotal study 1. Once the plasma total phenytoin C_max_ concentration was higher than 6,000 ng/mL or the AUC_0–4h_ reached 1,800 ng h/mL, the incidence of neurological symptoms, such as dizziness, headache, and hypesthesia, increased.

## 4 Discussion

This study carried out a comparison of the intravenous and intramuscular bioavailability and safety between CE-fosphenytoin sodium (test drug, T) and fosphenytoin sodium (Cerebyx^®^), which is an equivalent dose of a commercially available reference product (R1). Meanwhile, phenytoin sodium was also tested as a reference drug (R2) for comparison in the administration (i.v.) study. There were neither SAEs nor grade III AEs during the period of treatment with T and R1, suggesting that both T and R1 are safe and well-tolerated. With either intravenous or intramuscular administration, the incidence of the drug-related AEs in the T group was similar to that in the R1 group. The common adverse events of fosphenytoin sodium were dizziness, headache, pruritus, fatigue, and tremor, according to the drug label of Cerebyx^®^ and the reports of previous studies on fosphenytoin sodium. In this study, the adverse events that occurred after a single intravenous and intramuscular administration of T and R1 were similar to the most common occurred adverse events, as described previously.

All of the subjects (*n* = 18) in the R2 group experienced pricking pain at the injection site. Most of them also had dizziness and numbness, and one subject had grade III headache. However, the incidence rate of AEs as mentioned previously was much lower in the T and R1 groups. Some subjects in the R2 group had intolerable AEs in period 1; therefore, the subsequent administration of R2 was canceled, which was approved by the safety committee. It is obvious that both T and R1 had clearly superior tolerability, compared to R2.

In this study, we found similar pharmacokinetic characteristics between T and R1 when both drugs were administered by either i.v. or i.m. T and R1 were considered bioequivalent, and the point estimate was close to 1. In the i.v. study, the AUC of total and free phenytoin in the subjects who received T and R1 was very similar to those who received R2, although the C_max_ of total and free phenytoin in both the T and R1 groups was lower than that in the R2 group, because both T and R1 need to be converted to R2 in the body. Lower C_max_ reflected that fewer AEs occurred in the T and R1 groups compared to the R2 group.

In a study in Japan on the bioequivalence between R1 and R2 (through i.v. administration) (Inoue et al., 2013), the AUC of R1 was equivalent to that of R2, but the C_max_ values of R1 and R2 were significantly different. The C_max_ of R2 was higher than that of R1, showing the same trend that we have found in this study. In that study (Inoue et al., 2013), using the same dosage, the C_max_ of total and free phenytoin in the R1 group as well as the AUC in the R1 and R2 groups were similar to our findings. However, in our study, the C_max_ value of total and free phenytoin in the R2 group was higher than that reported by the previous study, perhaps due to the different infusion rates (40 mg/min vs. 8.3 mg/min). PK parameters were simulated at the same infusion rate (8.3 mg/min), and the simulated C_max_ of total and free phenytoin were also similar to those previously reported (Table 2), suggesting that the difference between our study and other studies might be caused by different infusion rates.

Before the pivotal study, the pilot study was designed to explore a safe and reasonable dosage. For the intravenous study, according to the prescribing information for phenytoin sodium published by the NMPA, the recommended dose for convulsions in adults is between 150 and 250 mg with a rate not exceeding 50 mg/min, and the daily maximum dose should be less than 500 mg (NMPA). The prescribing information for the product published by the FDA suggests an adult loading dose of 10–15 mg/kg for status epilepticus and non-emergent situations and at a rate not exceeding 50 mg/min (FDA, 2021b), while for fosphenytoin sodium injection, the FDA prescribing information suggests an adult non-emergent loading dose of 10–20 mg PE/kg, which may be administered either through i.v. or i.m. (FDA, 2021a). Additionally, Inoue et al. (2013) also used an intravenous dose of 250 mg PE fosphenytoin sodium and 250 mg phenytoin sodium to compare the bioavailability in Japanese subjects. In addition, this study also confirmed that after a single intravenous dose of fosphenytoin sodium at doses of 250–500 mg PE, the C_max_ of plasma total phenytoin increased proportionally with increasing dose. In addition, in the FDA prescribing information for fosphenytoin sodium, following the administration of single i.v. doses of 400–1200 mg PE fosphenytoin sodium, the mean maximum total phenytoin concentrations increase in proportion to the dose (FDA, 2021a). After comprehensive consideration, doses of 250 mg PE for CE-fosphenytoin sodium (T), 250 mg PE for fosphenytoin sodium (R1), and 250 mg for phenytoin sodium (R2) were selected. An infusion rate of 40 mg PE/min was ultimately chosen for both the pilot and pivotal i.v. studies.

For the intramuscular study, based on the FDA drug label for fosphenytoin sodium, the recommended adult non-emergent loading dose is 10–20 mg/kg, administered via either i.v. or i.m. (FDA, 2021a). Therefore, doses of 400 mg, 600 mg, and 1000 mg of CE-fosphenytoin sodium (T) were selected for the i.m. pilot study to explore the linear relationship among the different doses. However, the designed study was changed because the incidence of AEs in the 600 mg R2 group was significantly higher than that in the 400 mg R2 group. Due to safety considerations, the 1,000 mg R2 group was excluded. In the i.m. pivotal study, 400 mg CE-fosphenytoin sodium (T) and fosphenytoin sodium (R1) were selected. The intramuscular administration of 1000 mg of T has been studied in the United States. In our study, we found that the C_max_ and AUC_0-t_ values of either 400 mg or 600 mg of R2 in Chinese subjects were basically linear with those in Americans who received 1,000 mg R2 (the data were not published).

## 5 Conclusion

Our study showed that treatment with T and R1 was safe and well-tolerated, and the incidence of the drug-related AEs in the T group was similar to that in the R1 group when they were either intravenously or intramuscularly administered. Both T and R1 had higher tolerability than R2. Similar pharmacokinetic characteristics for total and free phenytoin were found in T and R1 groups, both of which were bioequivalent in either i.v. or i.m. administration. In the i.v. study, the AUC of total and free phenytoin in the T and R1 groups was very similar to that in the R2 group, although the C_max_ of total and free phenytoin in both the T and R1 groups was lower than that in the R2 group, because both T and R1 need to be converted to R2 in the body.

## Data Availability

The raw data supporting the conclusion of this article will be made available by the authors, without undue reservation.
